# Identification and biochemical characterisation of tyrosine aminotransferase from *Anthoceros agrestis* unveils the conceivable entry point into rosmarinic acid biosynthesis in hornworts

**DOI:** 10.1007/s00425-021-03623-2

**Published:** 2021-04-12

**Authors:** Tobias Busch, Maike Petersen

**Affiliations:** Philipps-Universität Marburg, Institut für Pharmazeutische Biologie und Biotechnologie, Robert-Koch-Str. 4, 35037 Marburg, Germany

**Keywords:** Amino acid metabolism, Anthocerotopsida, Aromatic aminotransferase, Bryophytes, Hornworts, Ketoacid, 2-Oxoacid, Pyridoxal 5’-phosphate (PLP), Rosmarinic acid (RA), Transamination, Tyrosine aminotransferase (TAT)

## Abstract

**Main conclusion:**

Tyrosine aminotransferase (AaTAT) from the hornwort *Anthoceros agrestis* Paton (Anthocerotaceae) was amplified and expressed in *E. coli*. The active enzyme is able to accept a wide range of substrates with distinct preference for l-tyrosine, therefore, possibly catalysing the initial step in rosmarinic acid biosynthesis.

**Abstract:**

The presence of rosmarinic acid (RA) in the hornwort *A. agrestis* is well known, and some attempts have been made to clarify the biosynthesis of this caffeic acid ester in lower plants. Parallel to the biosynthesis in vascular plants, the involvement of tyrosine aminotransferase (EC 2.6.1.5; TAT) as the initial step was assumed. The amplification of a nucleotide sequence putatively encoding AaTAT (Genbank MN922307) and expression in *E. coli* were successful. The enzyme proved to have a high acceptance of l-tyrosine (*K*_m_ 0.53 mM) whilst slightly preferring 2-oxoglutarate over phenylpyruvate as co-substrate. Applying l-phenylalanine as a potential amino donor or using oxaloacetate or pyruvate as a replacement for 2-oxoglutarate as amino acceptor resulted in significantly lower catalytic efficiencies in each of these cases. To facilitate further substrate search, two methods were introduced, one using ninhydrin after thin-layer chromatography and the other using derivatisation with *o*-phthalaldehyde followed by HPLC or LC–MS analysis. Both methods proved to be well applicable and helped to confirm the acceptance of further aromatic and aliphatic amino acids. This work presents the first description of a heterologously expressed TAT from a hornwort (*A. agrestis*) and describes the possible entry into the biosynthesis of RA and other specialised compounds in a so far neglected representative of terrestrial plants and upcoming new model organism.

**Supplementary Information:**

The online version contains supplementary material available at 10.1007/s00425-021-03623-2.

## Introduction

Aromatic aminotransferases, an important class of pyridoxal-5′-phosphate (PLP)-dependent enzymes, catalyse the reversible transamination of aromatic amino acids, like l-tyrosine and l-phenylalanine, to form the corresponding 2-oxoacid and vice versa. The amino group is transferred to a 2-oxoacid (e.g. 2-oxoglutarate, oxaloacetate) which leaves the reaction as amino acid, while the incoming amino acid is transformed to the corresponding 2-oxoacid (e.g. 4-hydroxyphenylpyruvate, phenylpyruvate). The two subunits of the homodimeric aminotransferase each consist of one large and one small domain. The catalytic centre is formed at the interface of the two domains and the subunit interface (Hirotsu et al. 2005). PLP is covalently bound to the active site of the enzyme and requires a conserved Lys-residue whose ɛ-amino group forms an internal aldimine with PLP (Kirsch et al. 1984). By displacement by the reacting amino acid, the external aldimine is formed. Eventually, the 2-oxoacid is obtained by splitting off the α-proton and subsequent hydrolysis. Pyridoxamine-5′-phosphate remains bound to the enzyme and is regenerated in the second reaction phase by the accepting 2-oxoacid, which in turn is converted into the corresponding amino acid. Mechanistically, the entire process is therefore classified as a ping-pong bi-bi mechanism (Frey and Hegeman 2007). Aminotransferases in general can be separated into four subgroups, where aromatic aminotransferases are located in subgroup I together with aminotransferases transaminating aspartate or alanine (Mehta et al. 1993). Aminotransferases usually have a promiscuous character. Wang and Maeda (2018) demonstrate this in an overview of the already determined biochemical properties of aromatic aminotransferases found in various plant species. Therefore, biochemical assays to qualify the putative aminotransferase have to be performed in any case.

Aromatic aminotransferases are common enzymes in almost all living organisms, but with varying main functions. Bacterial and fungal aromatic aminotransferases are predominantly involved in the formation of the aromatic amino acids l-tyrosine and l-phenylalanine by transamination of 4-hydroxyphenylpyruvate (pHPP) and phenylpyruvate, respectively, and thus are main contributors to aromatic amino acid biosynthesis. The biosynthetic precursor of phenylpyruvate and pHPP is prephenate, which is derived from chorismate provided by the shikimate pathway (Gelfand and Steinberg 1977; Whitaker et al. 1982; Iraqui et al. 1998). In animals, since they are lacking the shikimate pathway and therefore essentially require aromatic amino acids as part of their diet, aromatic aminotransferases are solely involved in catabolic processes. Deficiency of tyrosine aminotransferase is known to cause type II tyrosinaemia (Hühn et al. 1998). Plants, with the exception of Fabaceae, which possess a cytosolic prephenate dehydrogenase involved in pHPP accumulation (Schenck et al. 2015), favour a divergent pathway, in which the formation of l-tyrosine and l-phenylalanine is determined by prephenate aminotransferase in plastids which catalyses the formation of l-arogenate from prephenate. The following reaction involves arogenate dehydrogenase to form l-tyrosine or arogenate dehydratase to form l-phenylalanine, respectively (Maeda and Dudareva 2012). Since bryophytes have only gradually moved into focus in recent years, there is, to our current knowledge, not yet sufficient evidence on their (aromatic) amino acid biosynthesis.

In plants, aromatic aminotransferases are primarily located in the cytosol and are, with the exceptions as indicated above, less important for aromatic amino acid biosynthesis (Yoo et al. 2013; Wang et al. 2016). In contrast to bacteria and fungi, they are mainly involved in the degradation of amino acids to 2-oxoacids. The intermediate pHPP formed from l-tyrosine contributes to the biosynthesis of primary compounds like plastoquinones and tocopherols and some specialised metabolites. The biosynthesis of ubiquinone, although structurally related to plastoquinone has not yet been comprehensively elucidated but features a different biosynthesis. Whereas l-phenylalanine contributes half to its formation by providing the intermediate 4-hydroxybenzoate, a second, l-tyrosine-led route can be assumed based on experiments with ^13^C-labeled l-tyrosine, by demonstrating the incorporation into the benzoquinone ring (Block et al. 2014; Liu and Lu 2016). The potential involvement of tyrosine aminotransferase (TAT), however, is not yet evident to our knowledge. This is quite different for tocopherols and plastoquinones, where transamination of l-tyrosine to pHPP and subsequent conversion to homogentisate, the shared ring precursor, through 4-hydroxyphenylpyruvate dioxygenase is well known (Xu et al. 2019). A direct correlation has been presented in *Arabidopsis thaliana* as TAT knockout mutants revealed reduced levels of tocopherols (Riewe et al. 2012; Wang et al. 2019). Since those are known for efficiently scavenging reactive oxygen species, it is no surprise that tocopherol levels can be induced by stress factors like age, wounding, (UV) light as well as regulators like jasmonate, methyl-12-oxophytodienoic acid, the herbicide oxyfluorfen and the toxin coronatine (Sandorf and Holländer-Czytko 2002; Holländer-Czytko et al. 2005). The involvement of TAT in the biosynthesis of benzylisoquinoline alkaloids has been suggested by Lee and Facchini (2011), who demonstrated a modest correlation between TAT transcript levels and benzylisoquinoline alkaloid accumulation through virus-induced gene-silencing in *Papaver somniferum* but noted that an additional source of the intermediate 4-hydroxyphenylacetaldehyde is plausible. This was substantiated by Torrens-Spence et al. (2018), who proposed the direct conversion of l-tyrosine to 4-hydroxyphenylacetaldehyde in parallel to salidroside biosynthesis. The situation is clearer regarding rosmarinic acid (RA). Studies on the importance of TAT as an entry-point enzyme in the tyrosine-derived part of RA biosynthesis have been conducted by De-Eknamkul and Ellis (1987a) and Mizukami and Ellis (1991) in *Anchusa officinalis* and more recently by Huang et al. (2008) in *Salvia milthiorrhiza*, Lu et al. (2013) in *Perilla frutescens*, Kim et al. (2014) in *Scutellaria baicalensis* and Ru et al. (2017) in *Prunella vulgaris*.

Rosmarinic acid, formally an ester of caffeic acid and 3,4-dihydroxyphenyllactic acid, is well known for its biological activities, like anti-inflammatory, antioxidant, antibacterial and antiviral effects (Amoah et al. 2016). Whereas the caffeic acid moiety is derived from l-phenylalanine through the phenylpropanoid pathway, the 3,4-dihydroxyphenyllactic acid part is based on l-tyrosine and requires transamination to pHPP as initial step (Ellis and Towers 1970). The widely accepted biosynthetic pathway for RA has been elucidated in *Coleus blumei* (syn.: *Solenostemon scutellarioides*, *Plectranthus scutellarioides,* Lamiaceae) as model for higher plants (Petersen et al. 1993).

Since the first description of RA in *Rosmarinus officinalis* (Scarpati and Oriente 1958) it has been detected in a wide range of plant families with focus on Lamiaceae, sub-family Nepetoideae, and Boraginaceae but also in more distant as well as evolutionary lower plants, like monocotyledons and the fern family Blechnaceae (Petersen et al. 2009). The hornwort *A. agrestis* (Anthocerotaceae) is the first non-vascular plant with RA, its occurrence as well as findings of the corresponding glucoside and other related phenolics, e.g. anthocerotonic and megacerotonic acids, are remarkable (Takeda et al. 1990; Trennhäuser 1992; Vogelsang et al. 2005), especially as further discoveries in its assumed algal predecessors and the related “bryophytes” (liverworts, mosses) have not been reported (Petersen et al. 2009).

In comparison to mosses and liverworts, hornworts have gained only limited interest until Szövényi et al. (2015) established *A. agrestis* as model organism and opened up a new approach for future research on hornworts by publishing the first genome sequence. Hornworts feature a haploid, dominant gametophyte developing the firmly anchored horn-like sporophyte as part of generational change (Glime 2020). It often has symbiotic relationships, commonly with cyanobacteria of the genus *Nostoc*, and shows stomata and chloroplasts with pyrenoids (Villarreal and Renner 2012; Villarreal and Renzaglia 2015). In consequence of its multifaceted characteristics, it is seen as an important connecting group between green algae and tracheophytes and its phylogenetic placement was vigorously discussed. Newer insights into phylogenomics place hornworts as a sister phylum to liverworts and mosses and consider bryophytes as monophyletic (Puttick et al. 2018; Li et al. 2020; Zhang et al. 2020).

Due to its wide distribution in the plant kingdom, RA is usually unsuitable as biomarker, but yet, in this special case, the investigation of its biosynthesis may be important to understand the key enzymes at the crossroads of primary and secondary metabolism in a lower plant near to the threshold from water-living to land plants. Land plants developed a broad range of secondary metabolites with a large proportion being derived from the phenylpropanoid pathway, e.g. lignin, flavonoids, phenolic compounds, with the exception of some derivatives already found in streptophyte algae (de Vries et al. 2017). Better understanding of the evolution of phenylpropanoid metabolism as well as of associated derivatives, e.g. RA, can help to track the adaption of the green lineage to the land. Most recently, progress has been made in the characterisation of phenylpropanoid-associated enzymes in *A. agrestis* when Wohl and Petersen (2020a, 2020b) published the characterisation of cinnamic acid 4-hydroxylase and 4-coumarate CoA-ligase. In addition, the characterisation of any key enzyme enables a comparison to vascular plants and helps to clarify whether the capacity to synthesise RA is the result of a parallel development in different plant groups or whether hornworts are the first representatives and passed it on to evolutionary higher plant taxa. Our research targets the molecular and biochemical characterisation of *A. agrestis* TAT. We here describe the successful amplification of a TAT nucleotide sequence (Genbank MN922307) and heterologous expression of active protein in *E. coli*. The enzyme was biochemically characterised and screened for potential substrate promiscuity. This involves the enzyme’s ability to accept the 2-oxoacid prephenate.

## Materials and methods

### Plant material

A callus culture of *A. agrestis* (syn. *A. crispulus*) was kindly provided by Professor Binding (Binding and Mordhorst 1991) and a suspension culture was established in a hormone-free CB medium (Petersen 2003).

### Isolation of RNA and preparation of cDNA

*Anthoceros agrestis* suspension-cultured cells were separated from the medium after a four-day growth phase by vacuum filtration. mRNA isolation was performed using the acid guanidinium thiocyanate-phenol–chloroform extraction (Chomczynski and Sacchi 1987). RNA samples were checked for their integrity electrophoretically. Synthesis of complementary DNA (cDNA) for PCR was performed with the qScript cDNA SuperMix (Quantabio). For RACE-PCR the SMARTer^®^ RACE 5′/3′ Kit (Takara) was used to synthesise RACE-ready cDNA.

### PCR amplification and transformation of *E. coli* SoluBL21

Sequence alignments of *Plectranthus scutellarioides* TAT (Acc. No. AJ458993), *A. punctatus* (scaffold *Ap*6389, kindly provided by Prof. Steven Kelly, Department of Plant Sciences, University of Oxford) and *A. agrestis* (scaffold *Aa*34867; Szövényi et al. 2015) unveiled conserved regions used to obtain first gene-specific primers. All primers were purchased from Eurofins Genomics. Primer sequences and detailed PCR conditions are shown in Suppl. Table S1. Amplification results were verified by electrophoresis on 0.7% agarose gels in TAE-buffer (40 mM Tris, 20 mM acetic acid, 1 mM EDTA). Agarose segments containing DNA of interest were extracted and purified with the NucleoSpin Gel and PCR clean-up kit (Macherey–Nagel). After ligation into pDrive (Qiagen) and transformation of *E. coli* EZ (Qiagen), the inserted DNA was sequenced by Microsynth Seqlab. The missing 5′- and 3′-ends of the putative TAT cDNA were amplified with the SMARTer^®^ RACE 5′/3′ Kit (Takara) using gene-specific primers (Suppl. Table S1). After ligation into pDrive, transformation of *E. coli* EZ, plasmid isolation and sequence verification, full-length amplification was performed with Phusion^®^ High-Fidelity DNA Polymerase (2 U/µl; NEB) and buffer (NEB), followed by ligation into pDrive and transformation of *E. coli* EZ. To prepare for homologous recombination using *E. coli* EZ, full-length sequence primers with overlapping regions to pET-15b (Novagen) were designed and the full-length sequence was amplified again (Jacobus and Gross 2015). For expression, pET-15b harbouring the full-length *AaTAT* sequence was introduced into *E. coli* SoluBL21 (Amsbio).

### Expression of AaTAT

For protein expression, the *E. coli* SoluBL21 cells containing pET-15b with *AaTAT* were incubated in terrific broth (TB) medium (Tartoff and Hobbs 1987) with ampicillin (100 µg/ml) at 37 °C and 220 rpm. Isopropyl β-d-1-thiogalactopyranoside (IPTG; final concentration 1 mM) was added at an OD_600_ of 0.4 and incubation was continued overnight at 25 °C, 220 rpm. Cells were harvested by centrifugation (4 °C, 3000*g*, 10 min) and resuspended in 4 ml per g bacterial cell weight 50 mM potassium phosphate buffer (KPi) pH 8.0. The cells were treated with 50 mg lysozyme for 30 min on ice, followed by ultrasonication (on ice, four times 30 s with intermittent cooling for 30 s, 0.5 cycles, 100% amplitude). The supernatant was collected after centrifugation (4 °C, 10,000*g*, 10 min). AaTAT was purified from the crude protein extract with help of the *N*-terminally attached 6xHis-tag via metal-chelate chromatography with Ni–NTA resin (Novagen). An empty disposable column was filled with 1 ml Ni–NTA resin and equilibrated with His-tag binding buffer (50 mM KPi pH 8.0, 10 mM imidazole, 300 mM NaCl). The crude extract, after adjusting NaCl and imidazole concentrations, was added and the flow-through discarded. After washing with 3 × 2 ml washing buffer (50 mM KPi pH 8.0, 20 mM imidazole, 300 mM NaCl) and discarding the flow-through, 3 × 1 ml elution buffer (50 mM KPi pH 8.0, 250 mM imidazole, 300 mM NaCl) was used to elute the His-tagged AaTAT protein which was desalted and transferred to 0.1 M KPi buffer pH 7.0 through PD-10 columns (GE Healthcare). Aliquots of the purified protein were stored until use at − 80 °C.

Protein concentration was determined according to Bradford (1976) with bovine serum albumin (BSA) as standard.

### SDS-PAGE, Western blot and immunodetection

Discontinuous SDS-PAGE was essentially performed according to Laemmli (1970). The gel was then either stained with Coomassie Brilliant Blue R250 or subjected to Western blotting basically as described by Mahmood and Yang (2012), but using the Towbin et al. (1979) buffer system. The expressed proteins were detected with mouse anti-6xHis-tag monoclonal antibodies (ThermoFisher, MA1-21,315) and goat anti-mouse secondary antibodies conjugated to alkaline phosphatase (Life Technologies, A16087). Nitro blue tetrazolium chloride (NBT)/5-bromo-4-chloro-3-indolyl-phosphate (BCIP) was used for staining essentially following standard protocols (https://www.sysy.com/protocols/westernblot-ap-detection) where the alkaline phosphatase coupled to the secondary antibody dephosphorylates BCIP followed by oxidation and indoxyl dimerization to dibromodichloroindigo. Nascent reduction equivalents reduce NBT to the corresponding formazan (Sambrook and Russell 2001). The presence of His-tagged protein is indicated by a mixture of the two insoluble pigments, resulting in purple staining.

### TAT assays for HPLC analysis

Standard assays were prepared with 1 M Tris/HCl buffer pH 9.0, 32 mM 2-oxoglutarate, 0.08 mM PLP and purified enzyme (or in some cases crude protein extract). The pre-incubated assays (30 °C) were started by adding 150 mM l-tyrosine (l-Tyr; dissolved in 0.5 M HCl) to a final concentration of 4.2 mM l-Tyr and were further incubated at 30 °C. The reaction was stopped with 50–100 µl 6 M HCl and extracted with 500 µl ethyl acetate twice. To examine analyte degradation, HCl was replaced by 50 µl 6 M KOH. After evaporation of the ethyl acetate, the residue was dissolved in 100 µl of the HPLC eluent. 20 µl of the sample was analysed by HPLC using an Equisil ODS column (250 × 4 mm with pre-column 20 × 4 mm, particle size 5 µm; Dr. Maisch GmbH) with 45% aqueous methanol, 0.01% phosphoric acid as eluent at a flow of 1 ml/min and detection at 283 nm (Kempin 1994). Due to the instability of the reaction product pHPP, further investigations were carried out photometrically (see below).

### Photometric activity assays

The photometric assays were performed as an endpoint determination. With l-Tyr as substrate the decomposition product of the reaction product pHPP, 4-hydroxybenzaldehyde, was detected photometrically at 330 nm (Diamondstone 1966). Phenylpyruvate, the product of l-phenylalanine (l-Phe), was detected at 320 nm (Ru et al. 2017). Standard assays consist of 1 M Tris/HCl buffer pH 8.5, PLP, an amino acid together with the appropriate 2-oxoacid as well as various dilutions of purified enzyme and were performed at 40 °C (for detailed compositions see Suppl. Table S2). Linearity with respect to incubation time was determined beforehand. Generally, assays were performed in three parallels repeated thrice.

### Assays for TLC analyses

To detect potential substrates and their products by TLC, different assays with a total volume of 250 µl containing 2 mM of the respective substrates l-Tyr, l-Phe, l-dihydroxyphenylalanine (l-DOPA), l-aspartate (l-Asp), l-tryptophan (l-Trp), l-alanine (l-Ala), l-serine (l-Ser) or prephenate, 0.1 M KPi buffer pH 8.5 and 32 mM 2-oxoglutarate were prepared. In the case of prephenate 2-oxoglutarate was substituted by l-glutamate. After pre-incubation at 40 °C, a mixture of purified enzyme and PLP was added, resulting in a total PLP concentration of 0.48 mM. After incubation for 0, 5, 15 and 45 min, the reaction was stopped by heating to 95 °C for 10 min. To obtain high amounts of the reaction product of prephenate (presumably arogenate), the reaction time was prolonged and some concentrations were optimised. After centrifugation (16,000*g*, 5 min) 2 µl of each sample was applied to a silica gel 60 F_254_ TLC plate together with reference compounds (l-Tyr, l-Phe, l-DOPA, l-Asp, l-Trp, l-Ala, l-Ser). To quantitatively purify the reaction product of prephenate, the full volume of the sample was purified by TLC. Plates were developed in 1-butanol/acetic acid/water (40 + 10 + 10) (Matheron and Moore 1973). After drying, the TLC plate was stained by spraying with 0.3% ninhydrin, 3% acetic acid in 1-butanol. TLC on cellulose plates was used to prepare samples for LC–MS analyses.

### Verification of TLC results by HPLC

Twenty-five µl of the assays with l-Tyr, l-Phe, l-DOPA, l-Asp, l-Trp, l-Ala and l-Ser were derivatised with 50 µl *o*-phthalaldehyde (OPA) solution (0.067% OPA, 2.5% methanol, 0.0125% 2-mercaptoethanol in borate buffer pH 8.8 (0.078 M H_3_BO_3_, 0.03 M Na_2_B_4_O_7_ × 10 H_2_0, 0.02 M NaCl). 20 µl of the derivatised assay was analysed by HPLC using a Luna C18(2) column (150 × 4.60 mm with pre-column, particle size 5 µm; Phenomenex) with 0.1 M Na_2_HPO_4_ in 25% methanol pH 6.75 as eluent and detection at 340 nm. This experimental design is largely based on ESA Application note 70-0160P.

### Enzyme assays for LC–MS analyses

To further identify the reaction product of AaTAT with prephenate after TLC, the stationary phase was scraped off an unstained plate at the same R_f_-value as stained after ninhydrin treatment; a second sample with significantly lower R_f_-value but within the TLC-track was collected from the same unstained plate as well, serving as negative control. The adsorbent was extracted with 250 µl water, intermitted by shaking and centrifugation (16,000*g*, 5 min) and then filter-sterilised. The water was evaporated in a vacuum centrifuge and the dried residues redissolved in up to 40 µl water. Additionally, prephenate assays incubated for up to 120 min were vacuum-dried directly after stopping and dissolved in up to 40 µl water. Prior to analysis on an Agilent 1260 HPLC system, samples were derivatised with OPA solution (0.27% OPA, 10% methanol, 0.05% 2-mercaptoethanol and borate buffer pH 8.8). LC was performed using the solvents *A* = 0.1% aqueous formic acid and *B* = 0.1% formic acid in acetonitrile with the following gradient: 0–40 min 5% *B* → 100%, 40–45 min 100% *B*, 45–55 min 100% *B* → 5% *B*, flow rate 0.25 ml/min at 20 °C on a Multospher 120 RP18 column (250 × 2 mm, 5 µm; CS-Chromatographie Service) with detection in a diode array detector at 190–400 nm and MS using a micrOTOF-Q III with ESI source, negative mode (Bruker Daltonics).

### Phylogenetic analysis

The phylogenetic analysis of several tyrosine aminotransferases was performed using 14 amino acid sequences: *Salvia miltiorrhiza* (ABC60050), *Prunella vulgaris* (AJW87632), *Perilla frutescens* (ADO17550), *Coleus blumei* (CAD30341), *Scutellaria baicalensis* (AIV98132 for SbTAT1 and AIV98133 for SbTAT2), *Arabidopsis thaliana* (NP_200208 for AtTAT1 and NP_198465 for AtTAT2), *Papaver somniferum* (ADC33123), *Selaginella moellendorffii* (XP_024536669), *Klebsormidium nitens* (GAQ85880), *Marchantia polymorpha* (OAE18929), *Physcomitrium patens* (XP_024361307), *A. agrestis* (MN922307, this paper). Additionally, amino acid sequences of distantly related aminotransferases were added: *A. thaliana* TrpAT (AAO63403), *A. thaliana* IPAAT (AAP68293), *A. thaliana* PrephenAT (Q9SIE1), *Oryza sativa* AlaAT (BAA77261), *A. thaliana* AlaAT (Q9SR86), *A. thaliana* Ala/GluAT (AAN62333), *Glycine max* AspAT (AAC50015), *A. thaliana* (mitochondrial) AspAT1 (P46643), *A. thaliana* (cytoplasmic) AspAT2 (P46645), *A. thaliana* (plastidic) AspAT5 (P46248), *Nicotiana tabacum* HistidinolAT (CAA70403), *Atropa belladonna* PheAT (AHN10104), *Rosa* ‘Yves Piaget’ PheAT (Hirata et al. 2012). The evolutionary history was inferred using the Maximum Likelihood method based on the JTT matrix-based model (Jones et al. 1992) in Mega 7 (version 7.0.26, Kumar et al. 2016).

## Results

### Nucleotide and amino acid sequence of AaTAT

An internal partial *TAT* sequence (508 bp) was amplified by PCR with primers deduced from a sequence alignment of *Plectranthus scutellarioides TAT* (acc. no. AJ458993) and two putative *TAT* scaffolds from *A. punctatus* (Ap6389) and *A. agrestis* (Aa34867) using reverse-transcribed RNA from *A. agrestis*. The resulting sequence had a high identity to the above-mentioned sequences. 3′- and 5′-RACE PCR was used to amplify the missing cDNA ends. A putative nucleotide sequence of *AaTAT* with a length of 1395 bp (submitted to Genbank as MN922307), encoding a protein of 464 amino acid residues, was successfully amplified. It should be noted that a potential shorter enzyme variant is conceivable, driven by a second ATG codon in the sequence. BLASTp analysis of the translated amino acid sequence showed 46–62% identity to various putative plant TATs, e.g. from the green alga *Klebsormidium nitens* (GAQ85880), the moss *Physcomitrium patens* (XP_024361307), the liverwort *Marchantia polymorpha* (OAE18929) as well as vascular plants like the lycophyte *Selaginella moellendorffii* (XP_024536669) or the angiosperm *Salvia miltiorrhiza* (ABC60050) from the family Lamiaceae. The identity (EMBOSS Needle) to the enzymatically characterised TAT from *Coleus blumei* (*Plectranthus scutellarioides*; Acc. No. CAD30341) was at 36.7% and the similarity at 53.3% (Biastoff 2003).

Conserved domain search (CDD; https://www.ncbi.nlm.nih.gov/Structure/cdd/wrpsb.cgi) revealed putative conserved regions, e.g. the homodimer interface, the PLP binding site and the catalytic Lys-residue, which is necessary to form the internal aldimine with PLP. The remaining conserved amino acids, essential for aminotransferases, are detectable as well (Suppl. Fig. S1). The number in bracket quotes the position in pig AspAT (Ovchinnikov et al. 1973), which can be seen as a blueprint for the aminotransferase enzyme family. Arg435 (386) is highly conserved in the family I aminotransferases since it is important to fix the substrate’s deprotonated α-carboxy group of amino acid or 2-oxoacid. Asp263 (222), which forms a hydrogen bond to N1 of PLP and stabilises protonation of nitrogen over the whole reaction, is retrievable in all sequences, as well as the already addressed Lys296 (258) (Kirsch et al. 1984; Mehta et al. 1989).

### Biochemical characterisation of AaTAT

The AaTAT protein synthesised in *E. coli* SoluBL21 was purified by metal chelate chromatography and analysed by SDS-PAGE and Western blotting; protein extracts from bacteria harbouring the same vector without the AaTAT encoding sequence were treated in the same way. The protein (calculated mass including the 6xHis-tag, 53.09 kDa) is visible on the polyacrylamide gel and detectable with His-tag specific antibodies on Western blots (Suppl. Fig. S2). In comparison, the empty vector control showed no visible His-tagged protein at the same molecular mass.

HPLC analyses of TAT assays with crude bacterial protein extract showed high catalytic activity for both, the empty vector control and protein from *AaTAT*-transformed cultures, whereas no activity for the empty vector control and high activity for the *AaTAT*-transformed cultures was shown with purified enzyme preparations. This reflects the ubiquitous character of aminotransferases since the bacterial crude extract contains both, endogenous bacterial and recombinant plant aminotransferases. Further assays were therefore performed with purified AaTAT protein preparations. Photometric assays with the purified AaTAT revealed a pH-optimum of pH 7.9–8.4, which is in line with various plant TATs (Prabhu and Hudson 2010; Lee and Facchini 2011; Wang et al. 2016), and a temperature optimum of 60 °C, which is considerably higher than for comparable plant TATs. De-Eknamkul and Ellis (1987b) and Prabhu and Hudson (2010) report that 50% activity loss was observed for 65 °C, 55 °C, 50 °C (TAT1, TAT2, TAT3) and 60 °C, respectively. Nevertheless, reports about distantly related heat-stable aminotransferases, e.g. tryptophan aminotransferase featuring an optimum of 55 °C (Tao et al. 2008) and prephenate aminotransferase with an even higher optimum at 70 °C (Bonner and Jensen 1985), are known as well.

To determine general kinetic data, photometric assays, as described in Suppl. Table S2 were established. For PLP, concentrations of up to 1.92 mM have been tested. The reaction time was set to 1 min after linearity had been ensured for the highest and lowest PLP concentrations. Because of the covalent character of the PLP-enzyme complex, PLP addition should not be necessary, but optimal activity was measurable at 0.24 to 0.96 mM PLP and decreased sharply after adding higher concentrations. To our knowledge, this effect has not been described in literature before. As a result, 0.48 mM PLP was used in the following experiments (Suppl. Fig. S3).

**Fig. 1:**
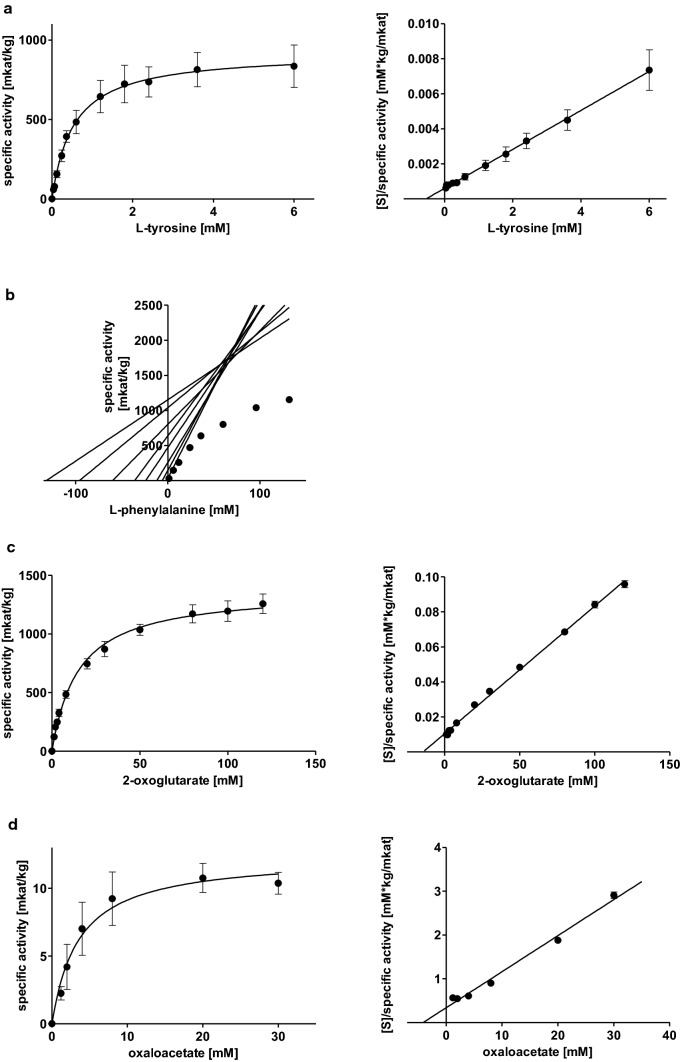
Michaelis–Menten and Hanes-Woolf plots of AaTAT activities for different substrate combination pairs: l-tyrosine with 2-oxoglutarate (**a**), l-phenylalanine with 2-oxoglutarate (**b**), 2-oxoglutarate with l-tyrosine (**c**), oxaloacetate with l-tyrosine (**d**). Means of 9 determinations ± SD, except l-Phe (*n* = 3 × 3), using three independent enzyme expressions

Substrate saturation curves for AaTAT with l-Tyr and l-Phe were determined with concentrations of up to 6 mM and 132 mM, respectively, together with 80 mM 2-oxoglutarate, after linearity was ensured for the highest and lowest administered substrate concentrations. l-Tyr was accepted with the highest affinity (*K*_m_ 0.53 ± 0.05 mM) and a *V*_max_ of 921.5 ± 142.5 mkat/kg (Fig. 1 a, Table 1). d-Tyrosine (d-Tyr) was not accepted, this was also confirmed by TLC (see below). l-Phe could not be added to the assays up to a saturating concentration, therefore kinetic data were analysed by direct linear plots according to Cornish-Bowden and Eisenthal (1978). To be able to compare results obtained with Michaelis–Menten equation and the linearization according to Cornish-Bowden and Eisenthal, a replicate of the l-Tyr kinetics with the number of measuring points adapted to l-Phe was also subjected to the calculation via Cornish-Bowden and Eisenthal. The results for l-Tyr (*K*_m_ 0.65 ± 0.13 mM and *V*_max_ of 998.9 ± 254.0 mkat/kg) are in accordance with the already mentioned parameters determined by Michaelis–Menten graphs. It can therefore be assumed that Cornish-Bowden plots can serve as an adequate substitute. The affinity of AaTAT towards l-Phe is 133-fold lower (*K*_m_ 70.47 ± 4.18 mM), but *V*_max_ was apparently much higher at 2240.3 ± 505.7 mkat/kg (Fig. 1b, Table 1). k_cat_/*K*_m_-values, however, indicated a more than 50 times higher kinetic efficiency for the use of l-Tyr.

**Table 1:** Kinetic parameters for AaTAT as means ± SD; all determinations are based on *n* = 9, except l-Phe (*n* = 3 × 3) using three independent enzyme expressions

Substrate 1	Substrate 2	*K*_m_ for substrate 1 [mM]	*V*_max_ [mkat/kg]	k_cat_ [1/s]	k_cat_/*K*_m_ [1/mM*s]
l-tyrosine	2-oxoglutarate	0.53 ± 0.05	921.5 ± 142.5	48.9 ± 7.6	91.7 ± 9.8
l-phenylalanine	2-oxoglutarate	70.47 ± 4.18	2240.3 ± 505.7	118.9 ± 26.9	1.7 ± 0.4
2-oxoglutarate	l-tyrosine	15.13 ± 1.34	1376.1 ± 97.6	73.1 ± 5.2	4.9 ± 0.3
Oxaloacetate	l-tyrosine	4.05 ± 1.44	12.6 ± 0.8	0.7 ± 0.04	0.2 ± 0.09
Phenylpyruvate	l-tyrosine	7.92 ± 2.35	530.1 ± 117.1	28.2 ± 6.2	3.9 ± 1.4
Pyruvate	l-tyrosine	81.14 ± 15.12	223.8 ± 30.0	11.9 ± 1.6	0.15 ± 0.02

The *K*_m_ and *V*_max_ values for l-Tyr, 2-oxoglutarate and oxaloacetate, phenylpyruvate and pyruvate are calculated from Michaelis–Menten equations, the K_m_ and V_max_ values for l-Phe were determined by Cornish-Bowden linear plots. k_cat_ was calculated assuming a molecular mass of 53.09 kDa for AaTAT including the 6xHis-tag

**Fig. 2:**
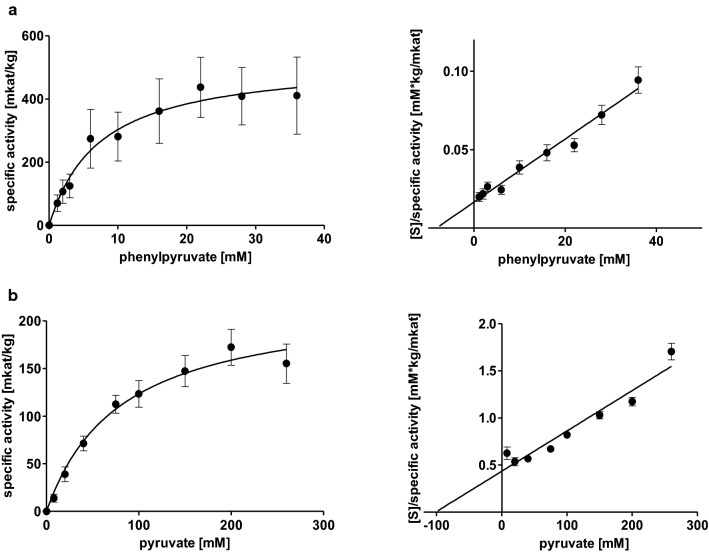
Michaelis–Menten and Hanes-Woolf plots of AaTAT activities with different substrate combination pairs: phenylpyruvate with l-tyrosine (**a**), pyruvate with l-tyrosine (**b**). Means of 9 determinations ± SD, using three independent enzyme expressions

AaTAT accepted not only 2-oxoglutarate as amino acceptor but also oxaloacetate, phenylpyruvate and pyruvate. To determine kinetic constants, 2-oxoglutarate and oxaloacetate were applied up to 120 mM each, phenylpyruvate up to 36 mM and pyruvate up to 260 mM, together with 6 mM l-Tyr, after ensuring linearity during the respective incubation time. The affinity for 2-oxoglutarate was lower but the activity was almost 100-fold (*K*_m_ 15.13 ± 1.34 mM, *V*_max_ 1376.1 ± 97.6 mkat/kg) compared to oxaloacetate which is accepted with the highest affinity of the examined amino acceptors (*K*_m_ 4.05 ± 1.44 mM) but with rather low activity (*V*_max_ 12.6 ± 0.8 mkat/kg) (Fig. 1 c and d, Table 1). Phenylpyruvate showed a lower activity (*V*_max_ 530.1 ± 117.1 mkat/kg) than 2-oxoglutarate but with a higher affinity (*K*_m_ 7.92 ± 2.35 mM) (Fig. 2 a, Table 1). Since the determined parameters for 2-oxoglutarate and phenylpyruvate were quite similar, a direct comparison was developed. It verified the higher substrate turnover of 2-oxoglutarate. Pyruvate was accepted but with a much higher *K*_m_-value (81.14 ± 15.12 mM) and mediocre turnover (*V*_max_ 223.8 ± 30.0 mkat/kg) (Fig. 2 b, Table 1). When comparing k_cat_/*K*_m_-values (Table 1), l-Tyr is the substrate converted most efficiently. On the side of the cosubstrate it is 2-oxoglutarate followed by phenylpyruvate.

### Search for other substrates

**Fig. 3:**
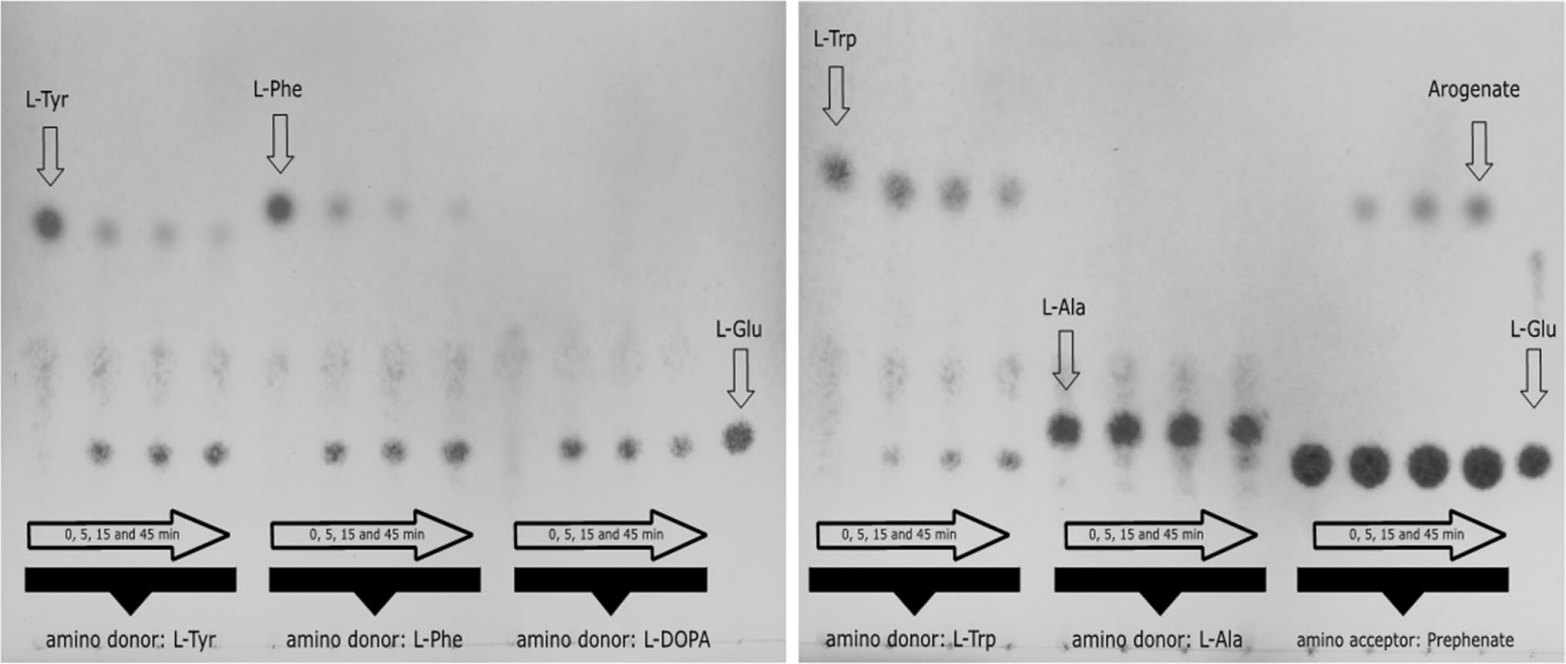
TLC of AaTAT assays using various putative substrates which turned out to be accepted by AaTAT. The five amino donors l-Tyr, l-Phe, l-DOPA, l-Trp and l-Ala (each with 2-oxoglutarate as amino acceptor substrate) as well as the amino acceptor prephenate with l-glutamate as amino donor substrate were incubated for 0, 5, 15 and 45 min. Glutamate is not detectable in 0 min assays. With increasing incubation time the substrate decreases and glutamate increases. Turnover rates can be estimated from the color differences. In contrast, the prephenate assays, as they are structured differently, show an increasing arogenate spot in the case of l-glutamate excess. The TLC plates were stained by spraying with 0.3% ninhydrin solution

**Fig. 4:**
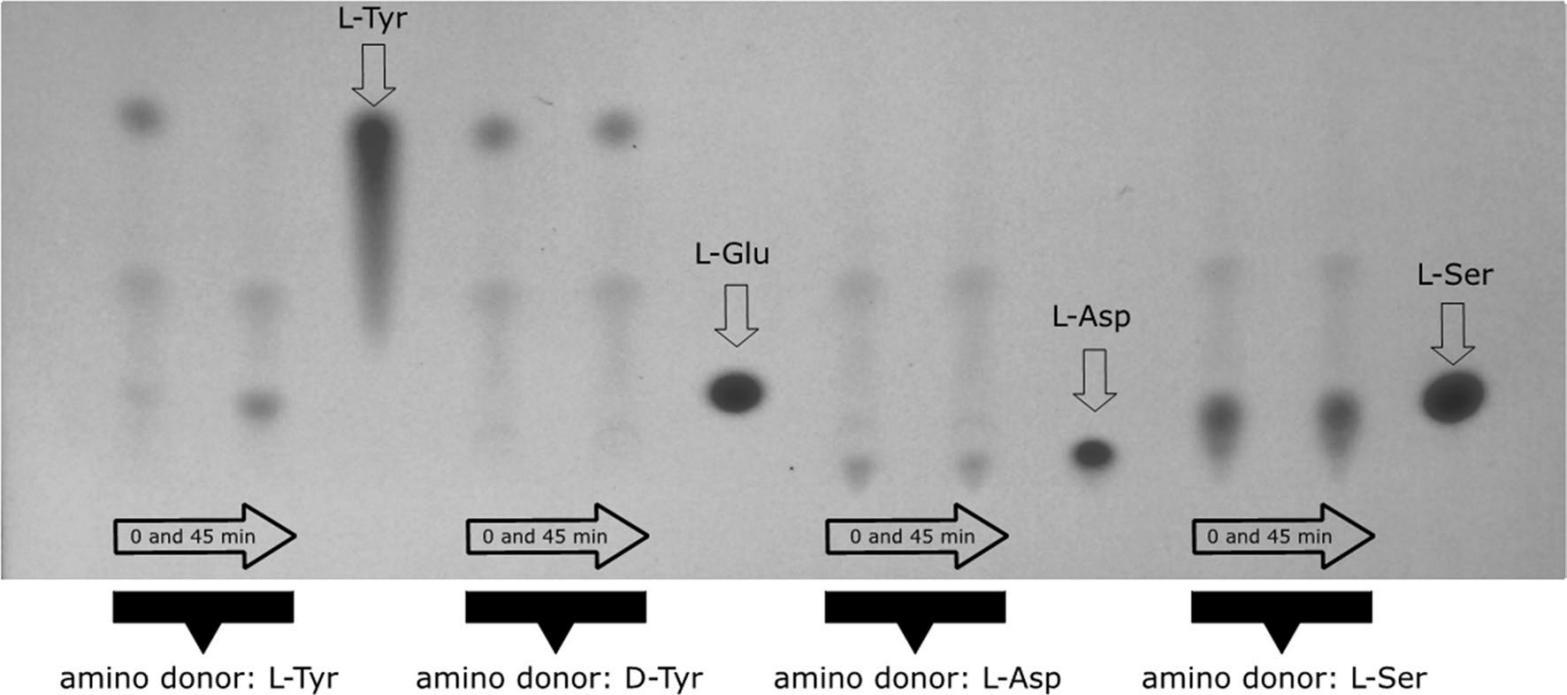
TLC of AaTAT assays using various putative substrates which failed to be accepted by AaTAT: d-Tyr, l-Asp and l-Ser as well as l-Tyr as a positive control. Each sample is shown after 0 min and 45 min incubation time. While the assay with l-Tyr leads to visible accumulation of glutamate, l-Tyr, l-Asp, and l-Ser show no glutamate formation. Relevant references for l-Glu, l-Asp, l-Ser are shown. The TLC plates were stained by spraying with 0.3% ninhydrin solution

Different alternative donor substrates and one further acceptor substrate were tested in AaTAT assays incubated for up to 45 min. Product formation was detected by TLC with subsequent visualisation by spraying with ninhydrin reagent. The analyses showed that l-Tyr, l-Phe, l-DOPA, l-Trp and l-Ala are accepted as substrates by AaTAT (Fig. 3) whereas l-Asp, l-Ser and d-Tyr are not accepted (Fig. 4). Evaluation of the colour depth (which correlates roughly with product formation) shows that l-Trp and l-Ala are less suitable substrates.

**Fig. 5:**
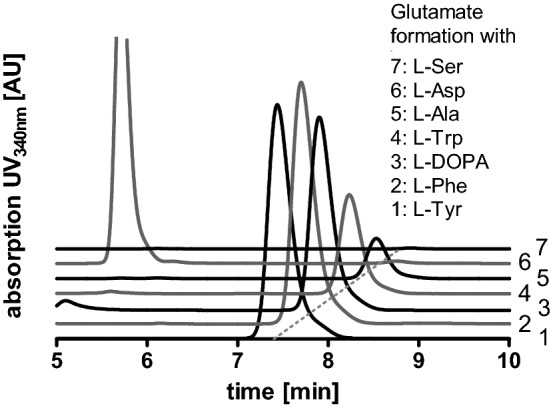
Verification of the TAT amino donor substrate acceptance profile by HPLC. The curves show the glutamate accumulation (after OPA derivatisation) when using the respective amino donor substrates. The donors l-Tyr, l-Phe, l-DOPA, l-Trp and l-Ala (1–5) are accepted, whereas no glutamate formation is observed for l-Asp (6) and l-Ser (7). The given area under the curve does not reflect the specific activity for the respective substrate

**Fig. 6:**
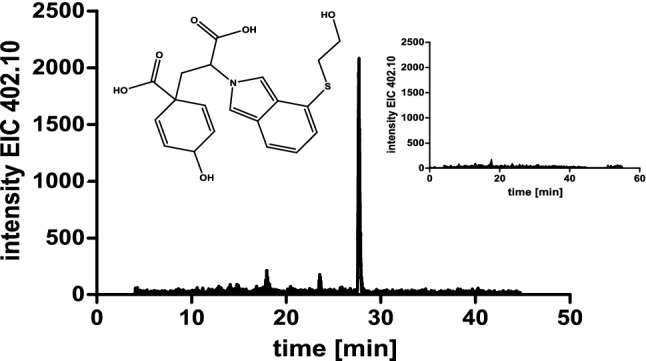
Identification of arogenate as OPA-derivative (extracted ion chromatogram for *m/z* 402.10 [M-H]^−^) vs. control (inset). The reaction product from assays using prephenate were extracted after TLC on cellulose plates

To verify the product formation when adding the above-mentioned donor substrates, samples were derivatised with OPA and analysed by HPLC (Fig. 5). The given area under the curve does not reflect the specific activity for the respective substrate, it only illustrates whether a substrate is accepted at all. The acceptance profile, previously determined using TLC (compare Figs. 3 and 4), was confirmed. Besides, TLC analysis showed product formation in assays with prephenate as amino acceptor together with l-glutamate as amino donor (Fig. 3). The expected product is arogenate. Since it is not available as standard, the reaction product of prephenate was purified by TLC and analysed as OPA-derivative by LC–MS (Fig. 6). The mass of the reaction product correlated to the mass of the OPA-derivative of arogenate.

A special feature of working with 2-oxoacids is their potential susceptibility to decarboxylation. Oxaloacetate is particularly noteworthy here (Tsai 1967). In enzyme assays containing oxaloacetate as amino acceptor and l-Tyr as amino donor, two reaction products could be detected on the TLC plates, l-Asp, as expected, and l-Ala as decarboxylated product (Suppl. Fig. S4).

## Discussion

Since the discovery of the involvement of TAT as an entry-point enzyme in RA biosynthesis (De-Eknamkul and Ellis 1987a), TAT activity has been studied in many plant species, e.g. *S. miltiorrhiza, A. thaliana* and *P. vulgaris* (Huang et al. 2008; Prabhu and Hudson 2010; Ru et al. 2017), all belonging to the vascular plants, but not all of these species synthesizing RA. In this study, we present AaTAT as the first molecularly and biochemically characterised enzyme connected to amino acid-derived specialised metabolism in hornworts, namely the biosynthesis of RA and related compounds. To our current knowledge, this is the evolutionarily lowest representative with this capacity (Petersen et al. 2009).

The AaTAT amino acid sequence complies with common aminotransferase characteristics and conserved amino acids (Suppl. Fig. S1), most importantly Arg435, indispensable for fixing the incoming carboxylate, Asp263, which is important to guarantee nitrogen protonation in PLP and Lys296, necessary to form the internal aldimine of PLP and the enzyme (Kirsch et al. 1984; Mehta et al. 1989). Furthermore, it resembles already characterised higher plant TATs. An alignment of the AaTAT amino acid sequence with such plant TATs, including those from *A. thaliana* (AtTAT1, AtTAT2), *P. vulgaris* (PvTAT), *S. miltiorrhiza* (SmTAT), *P. frutescens* (PfTAT), *S. baicalensis* (SbTAT1, SbTAT2), *Coleus blumei* (CbTAT) and *P. somniferum* (PsTAT), was used to visualise the similarities and differences to higher plants (Suppl. Fig. S1). As expected, PvTAT, SmTAT, PfTAT, and CbTAT, all members of the Lamiaceae, subfamily Nepetoideae, are highly similar. SbTAT1 and SbTAT2 (Lamiaceae, subfamily Scutellarioideae) already deviate in some areas and in length, but SbTAT1 shows higher agreement with the representatives of the Nepetoideae. The decrease in similarity with growing family distance continues with AtTAT1, AtTAT2 (Brassicaceae) and PsTAT (Papaveraceae). Differences between plant families are common, but interestingly AaTAT has an extended *N*-terminus (53 amino acid residues) compared to the representatives of the Nepetoideae, but no evidence for an *N*-terminal plastid signal peptide was found (SignalP-5.0; Almagro Armenteros et al. 2019). *Anthoceros agrestis* TAT shares this feature with putative TAT sequences of other representatives of the lower plants, e.g. *P. patens*, *K. nitens*, *M. polymorpha* and *S. moellendorfi* (sorted by decreasing *N*-terminal excess length). In AaTAT, the *N*-terminus has a second possible methionine start codon which gives the possibility for an additional shorter enzyme variant. The expression and activity determination of this shorter enzyme variant should be considered in the future.

**Fig. 7:**
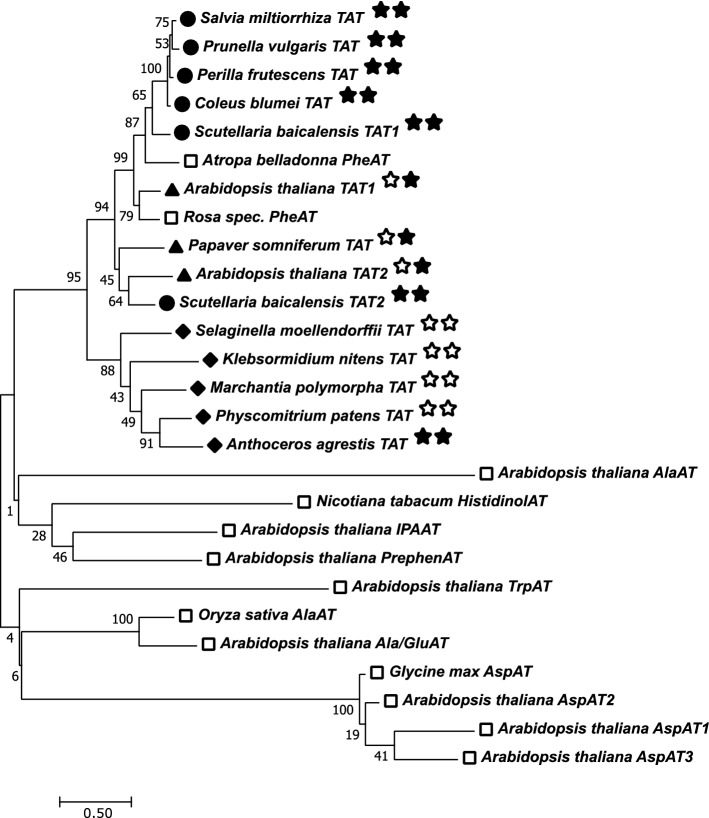
Phylogenetic tree (maximum likelihood analysis) of TAT amino acid sequences of several members of the Lamiaceae (circles) and representatives of other higher plant families (triangles) and lower plants (diamonds). Analyses were conducted in Mega 7 (Kumar et al. 2016). The tree with the highest log likelihood (− 13,478.93) is shown. The percentage of trees in which the associated taxa clustered together is shown next to the branches (bootstrap replicates 1000). Underlying TAT amino acid sequences: *Salvia miltiorrhiza* (ABC60050)*, Prunella vulgaris* (AJW87632)*, Perilla frutescens* (ADO17550)*, Coleus blumei* (CAD30341)*, Scutellaria baicalensis* (AIV98132 for SbTAT1 and AIV98133 for SbTAT2), *Arabidopsis thaliana* (NP_200208 for AtTAT1 and NP_198465 for AtTAT2), *Papaver somniferum* (ADC33123)*, Selaginella moellendorffii* (XP_024536669), *Klebsormidium nitens* (GAQ85880), *Marchantia polymorpha* (OAE18929), *Physcomitrium patens* (XP_024361307), *Anthoceros agrestis* (MN922307, this paper). The two stars following the name indicate the presence of RA in the plant (first star) and an experimentally proven TAT activity of the underlying protein (second star), if filled in. Underlying amino acid sequences of distantly related aminotransferases (open squares): *Arabidopsis thaliana* TrpAT (AAO63403), *A. thaliana* IPAAT (AAP68293), *A. thaliana* PrephenAT (Q9SIE1), *Oryza sativa* AlaAT (BAA77261), *A. thaliana* AlaAT (Q9SR86), *A. thaliana* Ala/GluAT (AAN62333), *Glycine max* AspAT (AAC50015), *A. thaliana* (mitochondrial) AspAT1 (P46643), *A. thaliana* (cytoplasmic) AspAT2 (P46645), *A. thaliana* (plastidic) AspAT5 (P46248), *Nicotiana tabacum* HistidinolAT (CAA70403), *Atropa belladonna* PheAT (AHN10104), *Rosa* ‘Yves Piaget’ PheAT (Hirata et al. 2012)

At sequence level, conserved regions, which are assigned to the PLP binding site and the homodimer interface, are, as previously mentioned, largely identical. But in addition, highly conserved regions in some areas outside of those essential domains are detectable, while the overall AaTAT amino acid sequence differs considerably from those of higher plant representatives. The phylogenetic tree (Fig. 7) gives an impression about the evolutionary relationship of TAT amino acid sequences from embryophytic plants. Several more distantly related subgroup I aminotransferases (Mehta et al. 1993), including tryptophan, alanine (and glutamate), aspartate, histidinol-phosphate, indole-3-pyruvate, prephenate and phenylalanine aminotransferases, are shown as well (open squares). It becomes evident that AaTAT has a close relationship to TATs of vascular plants and significantly lower consensus to the additional ones, except phenylalanine aminotransferases, likely because of the similarity of their substrate to tyrosine. This indicates a common origin. In the following, the more distantly related aminotransferases will not be discussed.

The diamond-marked major branch comprises TAT sequences from lower plants, including *A. agrestis*. The latter is the first enzymatically characterised species with respect to TAT. The second major branch is separated into Lamiaceae (circles) and the remaining dicotyledonous plants (triangles), where a presence of RA is not known. It illustrates the strong conservation within the Lamiaceae and indicates the probability of a specialised TAT for the RA biosynthetic pathway. Regarding TATs from non-Lamiaceae species, PsTAT is involved in alkaloid biosynthesis (Lee and Facchini 2011) and AtTAT1 and AtTAT2 are involved in tocopherol biosynthesis, with TAT1 having a higher contribution to l-tyrosine degradation (Riewe et al. 2012; Wang et al. 2019). Especially the differences in the two isoforms AtTAT1 and AtTAT2 suggest that there are relevant specialisations in amino acid metabolism. Two isoforms have been found for SbTAT as well, however, the exact function of these isoforms is still open (Kim et al. 2014). Other reports have already discussed several TAT isoforms and their deviating acceptance profiles for different amino acid and keto acid substrates (e.g. De-Eknamkul and Ellis 1987b for *Anchusa officinalis*; Wang et al. 2016 for *A. thaliana*). De-Eknamkul and Ellis (1987b) at this point already postulated a participation of TAT1 and to a lesser extent TAT2 in RA biosynthesis. In addition, they speculated that TAT2 may also be involved in plastoquinone or, even though a biosynthetic pathway unusual for plants, l-tyrosine biosynthesis.

However, searching for TAT sequences in *A. agrestis* databases always only revealed one isoform of TAT very similar or identical to the one described here while all other hits only displayed identities of less than 30%. The *A. punctatus* database contained potential additional TAT sequences (https://www.hornworts.uzh.ch/en/Blast.html). This makes a participation of AaTAT in RA biosynthesis probable, however, the exact biosynthetic route to l-tyrosine and l-phenylalanine in bryophytes has not yet been elucidated. Therefore, it is not known whether the biosynthesis of l-tyrosine runs via 4-hydroxyphenylpyruvate (as in bacteria and fungi) or via arogenate (as in higher plants). To get a more precise idea of this, it is essential to determine whether the formation or the degradation of l-tyrosine is favoured by AaTAT. In this respect, we can even only speculate about the acceptance of prephenate. Wang et al. (2016) were able to demonstrate the acceptance of prephenate for the TAT2 isoform in *A. thaliana*, the one that generally favours the conversion of pHPP to l-tyrosine and shows a similar broad acceptance profile.

In the next step, we further analysed biochemical characteristics and determined essential kinetic parameters for AaTAT (Table 1). SDS-PAGE visualises a protein of approximately 50 kDa (Suppl. Fig. S2), a range already reported for the TAT monomer in vascular plants (De-Eknamkul and Ellis 1987b; Prabhu and Hudson 2010). Most aromatic amino acid aminotransferases are not specific for only one substrate but accept a broader range of amino acids (Wang and Maeda 2018). Out of eight tested amino acids, six were accepted by AaTAT. Four of them are aromatic amino acids (l-tyrosine, l-phenylalanine, l-DOPA, l-tryptophan), the aliphatic amino acids accepted were l-alanine and l-glutamate, but not l-serine and l-aspartate. This is interesting since subgroup I aminotransferases also comprise aspartate and alanine aminotransferases (Mehta et al. 1993). While there are reports that describe active discrimination between aromatic and dicarboxylic amino acids, e.g. by providing complex hydrophobic interactions or offering charged and neutral pockets (Oue et al. 1997; Hirotsu et al. 2005), the acceptance of l-glutamate but not of l-aspartate, only differing by one CH_2_-group, was something we did not expect and which remains to be explained, especially, since the reverse reaction (oxaloacetate → aspartate), albeit with low turnover, is possible. Whether the discrimination against l-aspartate might be a feature of lower plant TATs has to be investigated for TAT from other algal or bryophyte sources. Since l-alanine is the simplest amino acid featuring a secondary amino group, it seems reasonable that the small nonpolar side chain fits into the binding pocket easily without change in conformation. l-Serine on the other hand is more demanding because of its slightly bigger and polar side chain.

When trying to compare these results with other publications, we come across various patterns. TAT1, TAT2 and TAT3 from *Anchusa officinalis* (De-Eknamkul and Ellis 1987b) are shown to accept aromatic amino acids with a distinct preference for l-tyrosine, but TAT2 and TAT3 also effectively accept l-glutamate and l-aspartate. Wang et al. (2016) report higher specificity of heterologously expressed TAT1 from *A. thaliana*, i.e. accepting aromatic amino acids whilst rejecting l-aspartate, and a broad substrate spectrum including l-aspartate for AtTAT2. Other reports mainly focus on aromatic amino acids and provide no information on the acceptance of aliphatic amino acids, or they focus on the 2-oxoacids (Prabhu and Hudson 2010; Lee and Facchini 2011; Riewe et al. 2012; Ru et al. 2017). Considering AaTAT, 2-oxoglutarate, phenylpyruvate, oxaloacetate and pyruvate could be utilised by AaTAT as amino acceptor, but 2-oxoglutarate is expected to be the main substrate. Additionally, prephenate was accepted leading to the amino acid arogenate as a reaction product. The efficiency of this reaction was, however, rather low. The acceptance of prephenate as 2-oxoacid has already been reported for other aromatic aminotransferases and seems not to be limited to prephenate aminotransferases (Wang et al. 2016).

The unambiguous characterisation of aromatic aminotransferases can therefore only be performed by individual kinetic characterisation and not just by sequence comparisons. We demonstrated high substrate specificity of AaTAT for the aromatic amino acid l-tyrosine, with lower acceptance of l-phenylalanine. The *K*_m_-value for l-tyrosine (0.53 ± 0.05 mM) is in line with recent publications: 1.82 ± 0.09 mM in *P. somniferum* (Lee and Facchini 2011), 0.19 ± 0.16 mM and 2.9 ± 0.4 mM, respectively, for TAT2 in *A. thaliana* (Prabhu and Hudson 2010; Wang et al. 2016), 0.18 ± 0.02 mM and 0.204 ± 0.001 mM, respectively, for TAT1 in *A. thaliana* (Riewe et al. 2012; Wang et al. 2016), 0.40 ± 0.05 mM in *P. vulgaris* (Ru et al. 2017). The affinity for l-phenylalanine is roughly 133-fold lower (*K*_m_ 70.47 ± 4.18 mM) for AaTAT. A similar relation (*K*_m_ for l-tyrosine 0.4 mM, *K*_m_ for Phe 10.2 mM) was found by Ru et al. (2017) for the heterologously expressed TAT from *P. vulgaris*. Hence, though accepting substrates promiscuously, AaTAT seems to be highly specific and can be defined as l-tyrosine aminotransferase. The outstanding *K*_m_/k_cat_-value for l-tyrosine underlines this assumption (Table 1). In conclusion, the obtained data suggest that the intracellular substrate/cosubstrate pair most probably is l-tyrosine/2-oxoglutarate. A role in RA biosynthesis is therefore quite conceivable.

Whether acceptance of additional amino acids has biological significance or not, remains to be clarified. But even l-phenylalanine, albeit structurally closely related to l-tyrosine, shows a significantly lower catalytic efficiency. To gain a more resilient understanding of AaTAT function, i.e. effects on primary and secondary metabolism in vivo, reverse genetics seem to be appropriate. Hücherig and Petersen (2013) already demonstrated that HPPR and RAS RNAi suppression in *Agrobacterium*-mediated *C. blumei* hairy root lines result in decreased levels of RA. Whether such a system is practicable for TAT without causing severe damage to the cells regarding its involvement in plastoquinone and tocopherol biosynthesis remains to be seen. Moreover, with preliminary evidence of phenylalanine ammonia-lyase (Pezeshki 2016) and full characterisation of cinnamic acid 4-hydroxylase and 4-coumarate CoA-ligase (Wohl and Petersen 2020a, 2020b), necessary enzymes of the general phenylpropanoid pathway in *A. agrestis* are already known. But since those enzymes as well as TAT cannot be assigned exclusively to RA biosynthesis, evidence for hydroxyphenylpyruvate reductase and RA synthase (RAS) still have to be provided to finally compare RA biosynthesis in *A. agrestis* and *C. blumei*. However, especially RAS, the key enzyme responsible for the esterification of 4-coumaroyl-/caffeoyl-CoA and 4-hydroxyphenyllactic/3,4-dihydroxyphenyllactic acid to the RA precursor (Petersen et al. 1993), has not yet been identified in *A. agrestis* with methods successful in Lamiaceae.

The highly discussed phylogeny of bryophytes ended in a commitment to the current research status that bryophytes can be seen as monophyletic clade, with hornworts as sister phylum to mosses and liverworts (Puttick et al. 2018; Li et al. 2020; Zhang et al. 2020). Since RA has been reported in hornworts but not in the related other two bryophyte groups and green algae (Petersen et al. 2009), it is possible that *A. agrestis* developed this capacity independently. This assumption may be supported by the possibility that ester formation is catalysed by an enzyme not belonging to the BAHD acyltransferase superfamily (Berger et al. 2006).

Since aromatic aminotransferases with their high substrate promiscuity have many potential amino donor/amino acceptor combinations, a full characterisation of these aminotransferases has rarely been performed and the substrates investigated are strongly dependent on the researchers’ intentions, including our work. The TLC/ninhydrin method we presented here can easily be used to screen potential aminotransferases for their substrate acceptance pattern and allows a cautious prediction of their respective activities. The sensitivity can be strongly increased by OPA derivatisation which is applicable for both analyses, i.e. HPLC and LC–MS. Using this method, we could even detect the only slight transformation of prephenate to arogenate. Photometric assays proved to be the first choice for the subsequent kinetic characterisation of the substrates. The mentioned methods or their variations have already been used by some researchers, but solely for specific applications. However, we here provide an example of how a combination of these approaches can be used to reliably clarify the acceptance profile of aminotransferases, estimate turnover and fully determine kinetic parameters. Remarkably, the methods verify each other and provide a full picture of the examined aminotransferase. This can serve to further research the biosynthesis of amino acid-based secondary metabolites in plants.

## Conclusion

The identification of TAT is an important milestone in the investigation of RA biosynthesis in *A. agrestis*. The enzyme was expressed successfully and showed a distinct affinity for l-tyrosine together with 2-oxoglutarate as amino acceptor. The reaction provides 4-hydroxyphenylpyruvic acid as substrate for further transformations in the direction to RA. Furthermore, acceptance of several aromatic and aliphatic amino acids as well as other possible amino acceptor substrates was shown.

### Author contribution statement

TB and MP conceived and designed the research. TB conducted the experiments, and TB and MP analysed the data and wrote the manuscript. Both authors read and approved the manuscript.

## Supplementary Information

Below is the link to the electronic supplementary material.

Supplementary file1 (PDF 1396 KB)

## Abbreviations

OPA: *o*-Phthtalaldehyde
pHPP: 4-Hydroxyphenylpyruvate
PLP: Pyridoxal-5′-phosphate
RA: Rosmarinic acid
(T)AT: (Tyrosine) aminotransferase
