# Supplementary material for: Identification and biochemical characterisation of tyrosine aminotransferase from Anthoceros agrestis unveils the conceivable entry point into rosmarinic acid biosynthesis in hornworts
Source: Planta. 2021 Apr 12;253(5):98. doi: 10.1007/s00425-021-03623-2 (PMC8041713; doi:10.1007/s00425-021-03623-2)
Supplement: Supplementary file 1 — Supplementary file1 (PDF 1396 KB) [file 425_2021_3623_MOESM1_ESM.pdf]

Supplementary Material for

Tobias Busch, Maike Petersen*

Identification and biochemical characterisation of tyrosine aminotransferase from *Anthoceros agrestis* unveils the conceivable entry point into rosmarinic acid biosynthesis in hornworts

Institut für Pharmazeutische Biologie und Biotechnologie, Philipps-Universität Marburg, Robert-Koch-Str. 4, D-35037 Marburg, Germany

* Corresponding author: petersen@staff.uni-marburg.de; telephone +49 6421 2825821

Supplementary Material and Methods

Suppl. Table S1 PCR primers and methods for amplification of partial and full-length *AaTAT* DNA sequences. The template cDNA was reverse-transcribed from RNA isolated from *Anthoceros agrestis* cell suspension cultures; F = forward primer, R = reverse primer; all primers were purchased from Eurofins Genomics

Method	Primers and assay composition	PCR program
Method 1:<br><br>Partial TAT sequence	F: GAGCACCTGCCGTTCGAG<br>R: CAAATGAGGATCCATCCCAACC<br><br>1 µl cDNA<br>5 µl 5X GoTaq buffer<br>0.5 µl 10 mM dNTPs<br>3 µl 25 mM $MgCl_2$<br>0.5 µl 10 µM gene specific primer F<br>0.5 µl 10 µM gene specific primer R<br>0.1 µl GoTaq Polymerase (5 U/µl, Promega)<br>PCR grade water ad 25 µl	Cycle 1: 94 °C 120 s, 54-60 °C 60 s, 70 °C 90 s<br>Cycle 2-40: 94 °C 30 s, 54-60 °C 60 s, 70 °C 90 s<br>Cycle 41: 94 °C 30 s, 54-60 °C 60 s, 70 °C 600 s
Method 2:<br><br>RACE-PCR	5'-RACE: CAGCTCCGCAACCTGCTCGAGGTCCACC<br>3'-RACE: TCGATCGCGTGCCTCGCGGCGGAAGG<br><br>Using Clontech SMARTer® RACE 5'/3' Kit<br>1.25 µl 5'- or 3'-RACE-Ready cDNA<br>12.5 µl 2X SeqAmp buffer<br>0.5 µl 10 µM 5'- or 3'-RACE primer<br>2.5 µl 10X Universal Primer Mix<br>0.5 µl SeqAmp DNA Polymerase<br>PCR grade water ad 25 µl	Cycle 1-5: 94 °C 30 s, 72 °C 180 s<br>Cycle 6-10: 94 °C 30 s, 70 °C 30 s, 72 °C 180 s<br>Cycle 11-37: 94 °C 30 s, 68 °C 30 s, 72 °C 180 s

Method	Primers and assay composition	PCR program
Method 3:<br><br>Full-length TAT cDNA	F: TATATCATATGGCCACAGTCCTGAACCAGAAGCG<br>R: ATAATCATATGCTAGTTTTGGACGGACGCATGTC GAAGAC<br><br>1 µl cDNA<br>5 µl 5X Phusion HF buffer<br>0.5 µl 10 mM dNTPs<br>0.5 µl 10 µM gene specific primer F<br>0.5 µl 10 µM gene specific primer R<br>1 µl Phusion Polymerase (2 U/µl, Thermo Fisher)<br>PCR grade water ad 25 µl	Cycle 1: 94 °C 120 s, 61-64 °C 60 s, 72 °C 90 s<br>Cycle 2-39: 94 °C 30 s, 61-64 °C 60 s, 72 °C 90 s<br>Cycle 40: 94 °C 60 s, 61-64 °C 60 s, 72 °C 300 s
Method 4:<br><br>Insert for homologous recombina-tion	F: GGTGCCGCGCGGCAGCCATATGGCCACAGTCCT GAACCAGAA<br>R: GGCTTTGTTAGCAGCCGGATCCTCGAGCTAGTT TTGGACGGACGCATGTC<br><br>1 µl full-length construct in pDrive<br>5 µl 5X Phusion HF buffer<br>0.5 µl 10 mM dNTPs<br>0.5 µl 10 µM gene specific primer F<br>0.5 µl 10 µM gene specific primer R<br>1 µl Phusion Polymerase (2 U/µl, Thermo Fisher)<br>PCR grade water ad 25 µl	Cycle 1: 94 °C 120 s<br>Cycle 2-40: 94 °C 30 s, 58 °C 30 s, 72 °C 90 s<br>Cycle 41: 72 °C 600 s

Suppl. Table S2 Detailed methods used for the determination of enzyme characteristics of AaTAT

Method	Assay
Method 1:<br><br>Determination of pH-optimum	Three different buffer systems were used to establish the pH optimum of AaTAT: range pH 4.0-10.0 – Britton-Robinson buffer (0.04 M phosphoric acid, 0.04 M acetic acid, 0.04 M boric acid titrated with 0.2 M NaOH (Britton and Robinson 1931), range pH 8.6-10.0 – 0.5 M *N*-cyclohexyl-2-aminoethanesulfonic acid (CHES) buffer titrated with 10 M NaOH; range pH 9.7-11.0 – 0.5 M *N*-cyclohexyl-3-aminopropanesulfonic acid (CAPS) buffer titrated with 10 M NaOH. Each assay was prepared in a total volume of 250 µl, consisting of the respective buffer, 32 mM 2-oxoglutarate, 0.08 mM pyridoxal phosphate (PLP) and purified enzyme. The pre-incubated assays (30 °C) were started by adding 150 mM L-tyrosine in 0.5 M HCl to a final concentration of 4.2 mM and further incubated at 30 °C for 1 minute. The reaction was stopped with 50 µl 6 M KOH and incubated at 45 °C for 30 minutes. References where prepared by stopping before substrate addition. Standards were prepared by substituting L-tyrosine by 4-hydroxyphenylpyruvate (pHPP). The samples were analysed photometrically against the reference at 330 nm. The actual pH-value was measured in each assay.
Method 2:<br><br>Determination of temperature optimum	To determine temperature-optimum, the range of 5.5 °C to 75 °C was tested. The assays were prepared and analysed based on method 1 using 1 M Tris-HCl buffer pH 8.5.

**Method**	**Assay**
Method 3: Determination of PLP dependence	To determine saturation curves for the prosthetic group PLP, concentrations of up to 1.92 mM PLP were tested. Each assay was prepared in a total volume of 250 µl consisting of 1 M Tris-HCl pH 8.5, 80 mM 2-oxoglutarate, 6 mM L-tyrosine and varying PLP-concentrations. After pre-incubation at 40 °C, the reaction was started by adding purified enzyme. The remaining reaction conditions are based on method 1.
Method 4: Substrate-saturation kinetics for L-tyrosine	To determine substrate-saturation curves for L-tyrosine, concentrations of up to 6 mM were tested. Each assay was prepared in a total volume of 250 µl consisting of 1 M Tris-HCl pH 8.5, 80 mM 2-oxoglutarate and varying L-tyrosine concentrations. After pre-incubation at 40 °C, a mixture of purified enzyme and PLP was added, resulting in a total PLP concentration of 0.48 mM. Incubation time was adjusted to 2 minutes. The remaining conditions are based on method 1.
Method 5: Substrate-saturation kinetics for 2-oxoglutarate	To determine substrate-saturation curves for 2-oxoglutarate, concentrations of up to 120 mM were tested. Each assay was prepared in a total volume of 250 µl consisting of 1 M Tris-HCl pH 8.5, 6 mM L-tyrosine and varying 2-oxoglutarate concentrations. After pre-incubation at 40 °C, a mixture of purified enzyme and PLP was added, resulting in a total PLP concentration of 0.48 mM. The remaining conditions are based on method 1.
Method 6: Substrate-saturation kinetics for oxaloacetate	To determine substrate-saturation curves for oxaloacetate, concentrations of up to 120 mM were tested. Each assay was prepared in a total volume of 250 µl consisting of 1 M Tris-HCl pH 8.5, 6 mM L-tyrosine and varying oxaloacetate concentrations. After pre-incubation at 40 °C, a mixture of purified enzyme and PLP was added, resulting in a total PLP concentration of 0.48 mM. Incubation time was adjusted to 2 minutes. The remaining conditions are based on method 1.
Method 7: Substrate-saturation kinetics for phenylpyruvate	To determine substrate-saturation curves for phenylpyruvate, concentrations of up to 36 mM were tested. Each assay was prepared in a total volume of 250 µl consisting of 1 M Tris-HCl pH 8.5, 6 mM L-tyrosine and varying phenylpyruvate concentrations. After pre-incubation at 40 °C, a mixture of purified enzyme and PLP was added, resulting in a total PLP concentration of 0.48 mM. Incubation time was adjusted to 1 minute. The remaining conditions are based on method 1. In order to optimise pH-value directly before measurement, 1 M Tris-HCl pH 7 was added.
Method 8: Substrate-saturation kinetics for pyruvate	To determine substrate-saturation curves for pyruvate, concentrations of up to 260 mM were tested. Each assay was prepared in a total volume of 250 µl consisting of 1 M Tris-HCl pH 8.5, 6 mM L-tyrosine and varying pyruvate concentrations. After pre-incubation at 40 °C, a mixture of purified enzyme and PLP was added, resulting in a total PLP concentration of 0.48 mM. Incubation time was adjusted to 0.5 minutes. The remaining conditions are based on method 1. In order to optimise pH-value directly before measurement, 1 M Tris-HCl pH 7 was added.
Method 9: Comparison of L-tyrosine and D-tyrosine	To compare the specific enzyme activities with L- and D-tyrosine, assays in a total volume of 250 µl consisting of 1 M Tris-HCl pH 8.5, 80 mM 2-oxoglutarate and 6 mM L- or D-tyrosine were prepared. After pre-incubation at 40 °C, a mixture of purified enzyme and PLP was added, resulting in a total PLP concentration of 0.48 mM. The remaining conditions are based on method 1.

Method	Assay
Method 10:<br><br>Substrate-saturation kinetics for L-phenylalanine	To determine substrate-saturation curves for L-phenylalanine, concentrations of up to 132 mM were tested. Each assay was prepared in a total volume of 250 µl consisting of 1 M Tris-HCl pH 8.5, 80 mM 2-oxoglutarate and varying L-phenylalanine concentrations. After pre-incubation at 40 °C, a mixture of purified enzyme and PLP was added, resulting in a total PLP concentration of 0.48 mM. The remaining conditions are based on method 1.

Supplementary Results

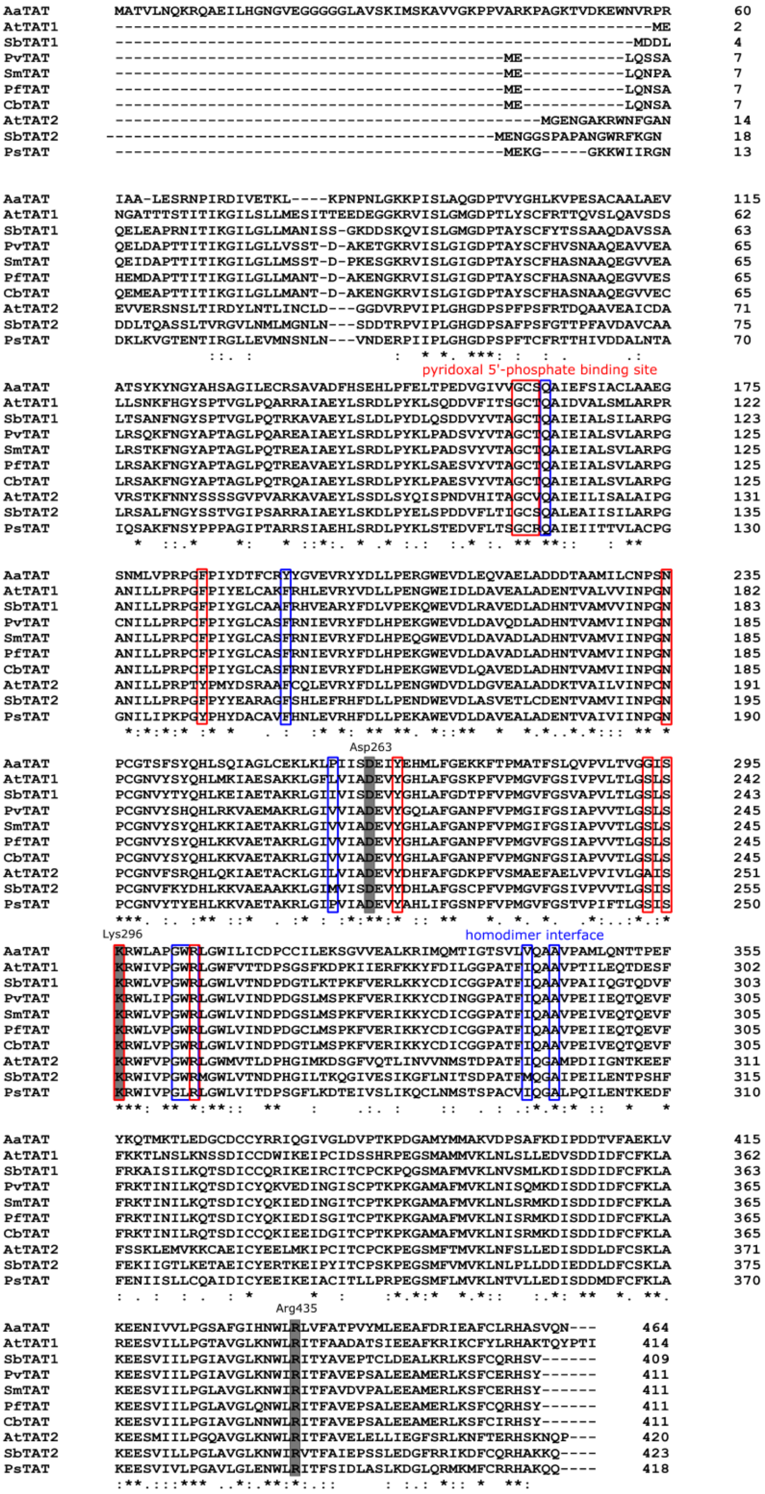

Suppl. Fig. S1 Alignment of some characterised plant tyrosine aminotransferases (*Scutellaria baicalensis* (AIV98132 for SbTAT1 and AIV98133 for SbTAT2), *Prunella vulgaris* (AJW87632), *Salvia milthiorrhiza* (ABC60050), *Perilla frutescens* (ADO17550), *Coleus blumei* (CAD30341), *Arabidopsis thaliana* (NP_200208 for AaTAT1 and NP_198465 for AaTAT2), *Papaver somniferum* (ADC33123) and *Anthoceros agrestis* (MN922307, this paper) TAT. Alignment was created with Clustal Omega (Madeira et al. 2019)

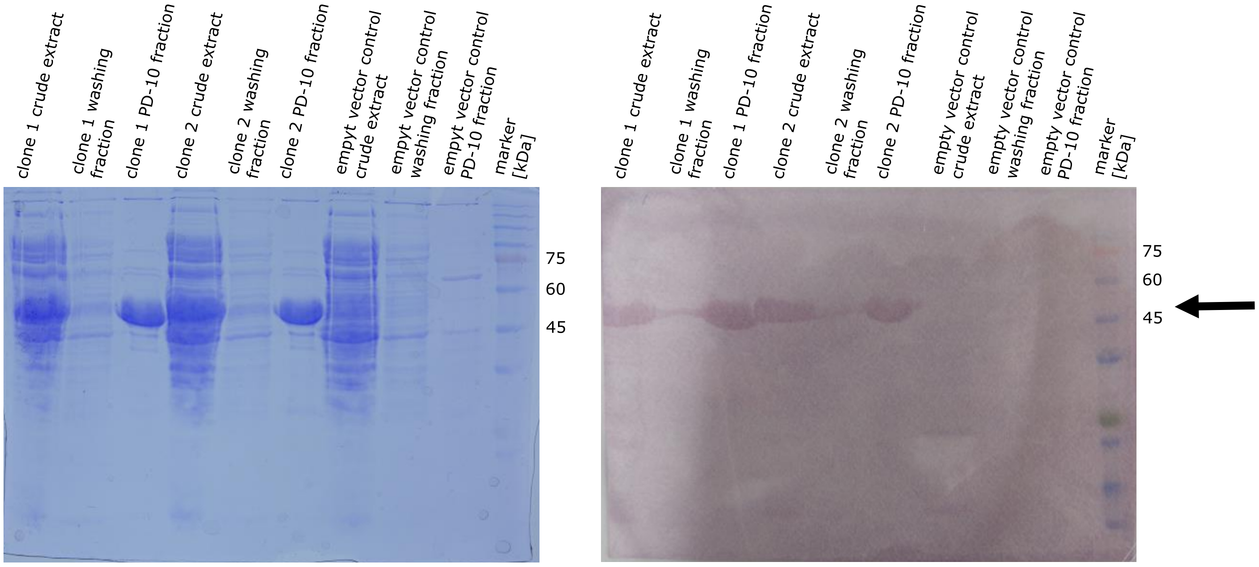

Suppl. Fig. S2 Purification and expression analysis of AaTAT (SDS-PAGE (left) and Western-Blot (right)). PD-10 fractions of both clones show distinct TAT bands at the estimated molecular weight vs. empty vector control (arrow)

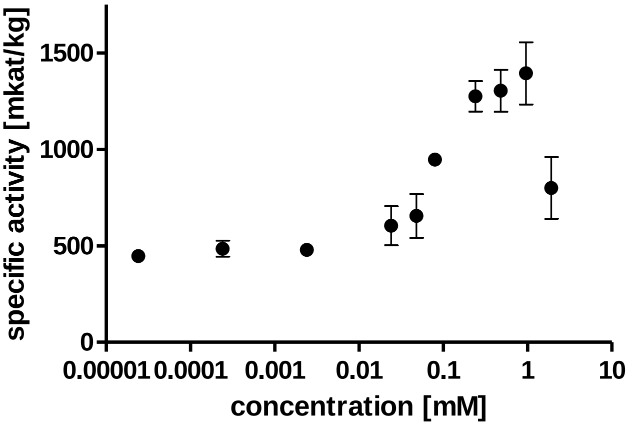

Suppl. Fig. S3 Impact of increasing PLP concentrations on TAT activity showing a plateau phase at 0.24-0.98 mM, abscissa in logarithmic scale

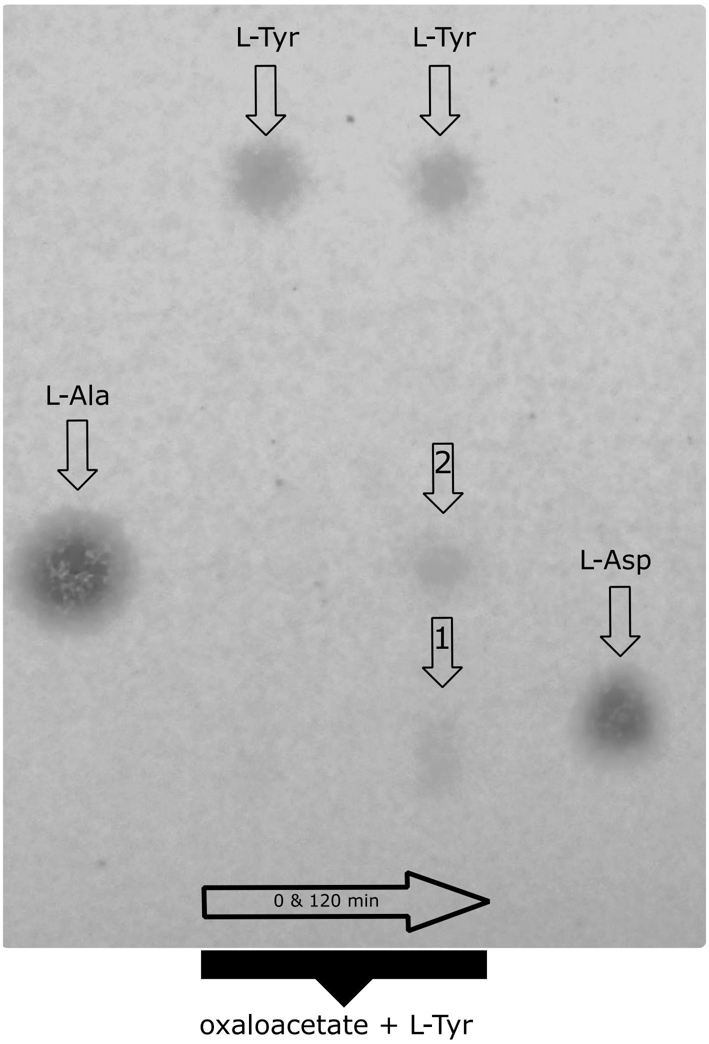

Suppl. Fig. S4 TLC analysis of the two potential reaction products of a TAT assay with oxaloacetate as amino acceptor and L-Tyr as amino donor. 1 represents the expected L-Asp (the slight shift in comparison to the reference results from the other assay components) and 2 represents L-Ala

Supplementary References

Britton HTS, Robinson RA (1931) CXCVIII. Universal buffer solutions and the dissociation constant of veronal. J Chem Soc 0:1456–1462. https://doi.org/10.1039/JR9310001456

Madeira F, Park YM, Lee J, Buso N, Gur T, Madhusoodanan N, Basutkar P, Tivey ARN, Potter SC, Finn RD, Lopez R (2019) The EMBL-EBI search and sequence analysis tools APIs in 2019. Nucl Acids Res 47:W636-W641. https://doi.org/10.1093/nar/gkz268
